# The Trend of Metaverse and Augmented & Virtual Reality Extending to the Healthcare System

**DOI:** 10.7759/cureus.29071

**Published:** 2022-09-12

**Authors:** Kunal Bhugaonkar, Roshan Bhugaonkar, Neha Masne

**Affiliations:** 1 Medicine, Jawaharlal Nehru Medical College, Datta Meghe Institute of Medical Sciences, Nagpur, IND; 2 Anesthesiology, Treat Me Hospital, Nagpur, IND

**Keywords:** medical education, healthcare, virtual reality, augmented reality, metaverse

## Abstract

There is no escaping Internet's favorite buzzword for 2022: The Metaverse. Everyone is talking about it, but only a few know what it is or how it works. One can look at the Metaverse as a 3D model of the Internet where it is possible to spend your reality parallel to the virtual world. In broad terms, Metaverse can be explained as a virtual space, graphically rich, leaning towards verisimilitude where people can do all sorts of things they do in real-life such as shop, play, socialize, and party. The pandemic has accelerated innovations in the digital age. Looking beyond revolutions in telehealth, payments, remote monitoring, and secure data-sharing are other essential innovations in the fields of artificial intelligence (AI), virtual reality (VR), augmented reality (AR), and blockchain technology. The Metaverse is still in its nascent stage and evolving continuously, having a huge potential in health care to combine the technologies of AI, AR/VR, web 3.0, Internet of medical devices, and quantum computing, along with robotics to give a new direction to healthcare systems. From improving surgical precision to therapeutic usage and more, the Metaverse can bring significant changes to the industry

## Introduction and background

Recent advances in VR technology have made it a very exciting and emerging field today [1]. In June 2020, neurosurgeons at Johns Hopkins University performed their very first AR surgery on a living patient. In the first procedure, physicians fixed six screws to fuse three vertebrae in the spine of a patient with severe debilitating back pain. In the second surgery, a cancerous tumor was removed from the patient's spine. During these surgeries, the surgeons wore headsets consisting of see-through eye displays which projected the images of the patient's interior anatomy based on already done computed tomography (CT) scans [2,3].

We are moving quickly towards the age of the Metaverse. The World Economic Forum already anticipates that one of the most revolutionary factors in transforming health care would be digital services [4]. With the COVID19 pandemic, telehealth went mainstream. Face-to-face contact was considered dangerous, and remote care became increasingly accepted [5]. Telepresence, digital twinning, and blockchain confluence are the three significant technical phenomena, according to futurist Bernard Marr, that have the potential to influence healthcare. These three ideas might be used to provide whole new methods of providing treatment, potentially reducing costs and significantly enhancing patient outcomes [6]. The greatest tech companies gradually began to aggressively engage in this previously unexplored region and assessed the numerous potential uses for the technology industry. Even Facebook formally changed its name to Meta, demonstrating its aspirational goal to become the dominant social media platform into a sizable Metaverse [7].

Metaverse includes the integration and overlapping of the digital and physical world, the integration of digital and real economies, the integration of digital and social life, the integration of digital and real identities, and the integration of digital with physical assets. It includes high-speed communication networks, the Internet of things (IoT), AR, VR, cloud computing, edge computing, blockchain, AI, and other technology. Technology is the driving factor that promotes the transition from the current Internet to Metaverse. The eight fundamental technologies are extended reality, user interaction (human-computer interaction), AI, blockchain, computer vision, IoT and robotics, edge and cloud computing, and future mobile network. There is still a big gap in achieving Metaverse transformation in the medical and health field. Existing platforms are still far from an ideal Health Metaverse, requiring the efforts of all parties [8]. Some of the well-known companies in the AR and VR market are Google, Microsoft, DAQRI, Psious, Mindmaze, Firsthand Technology, Medical Realities, Atheer, Augmedix, and Oculus VR [9].

It is expected that all these new concepts will greatly enhance comprehensive health care along with the prevention and treatment of diseases and will completely enhance the current model and usher in a new era in this industry. This review article sets out the viewpoint that VR/AR could be a new emphasis of direction in the development of training tools for medical education and communication skills for clinicians and medical students [10].

## Review

Digital world, digital patient

Medicine has always been a hands-on face-to-face personal experience. However, with the advent of newer technology, this is changing rapidly. It is true that AR and VR technologies have driven the gaming and entertainment industry, but it also has a very good potential to transform the healthcare industry since they can change a lot of traditional healthcare operations and branches in a variety of ways, including radiology, oncology, training, and more [11].

A field where AR/VR has proved to be particularly beneficial is therapy. Psychologists and psychiatrists use it to personalize environments for individual patients in aversion therapy, where patients interact with situations causing them anxiety in a controlled and safe environment where the interaction is monitored closely [12]. Surgical simulations, diagnostic imaging modality, patient care management, rehabilitative services, and healthcare management will be the earliest applications of the Metaverse. Patients can learn better about their disorders and treatment alternatives with this technology. In a clinical context, AR/VR can help nursing teams at the point of care. When AR is used with radiology, clinicians can display medical images, such as CT scan images, directly onto the patient and in arrangement with the patient's body, even when the person is moving, allowing them to examine interior anatomy more clearly [13].

Intravenous injections, for example, can benefit from technology like Accuvein's, which projects the map and plots the patient's own veins on their skin. Medtronic announced the acquisition of Digital Surgery, while Zimmer Biomet unveiled OptiVuTM Mixed Reality, employing HoloLens by Microsoft to blend the digital and real worlds. Through data interconnection, avatars will simulate realistic consultations, individualized treatment, diagnosis, and care [14]. Dimensional avatars of healthcare professionals will be able to interact with equipment like digital whiteboards in the Metaverse, and they will be able to make face-to-face contact without the use of complicated conference equipment. Before being used in a physical context, digital twins will be used to experiment and evaluate machines, systems, and procedures for flaws and vulnerabilities [15].

A digital twin can be described as a virtual model or simulation of any process, system, or object produced with real-world data in order to learn more about its real-world counterpart. The patient's digital twin may be the patient himself in the Metaverse. Individual digital twins will someday be utilized as test dummies to predict anything from surgery recovery to drug reactions. As our ability to map and understand individual DNA increases, this will become a reality [16]. Thanks to telemedicine consultations that particularly employ VR, patients will no longer be limited to being seen by a particular or specified doctor due to their present location. You can virtually be in the same room with the best specialist for your disease by simply putting on headsets even if you are physically on separate continents. Scans and testing can be done at your nearest physically accessible centers, and the results can be emailed to a specialist anywhere on the planet. It is especially useful in remote locations where medical personnel are scarce, and for patients in desolate areas who would otherwise have to travel vast distances to see a doctor [16].

**Figure 1:**
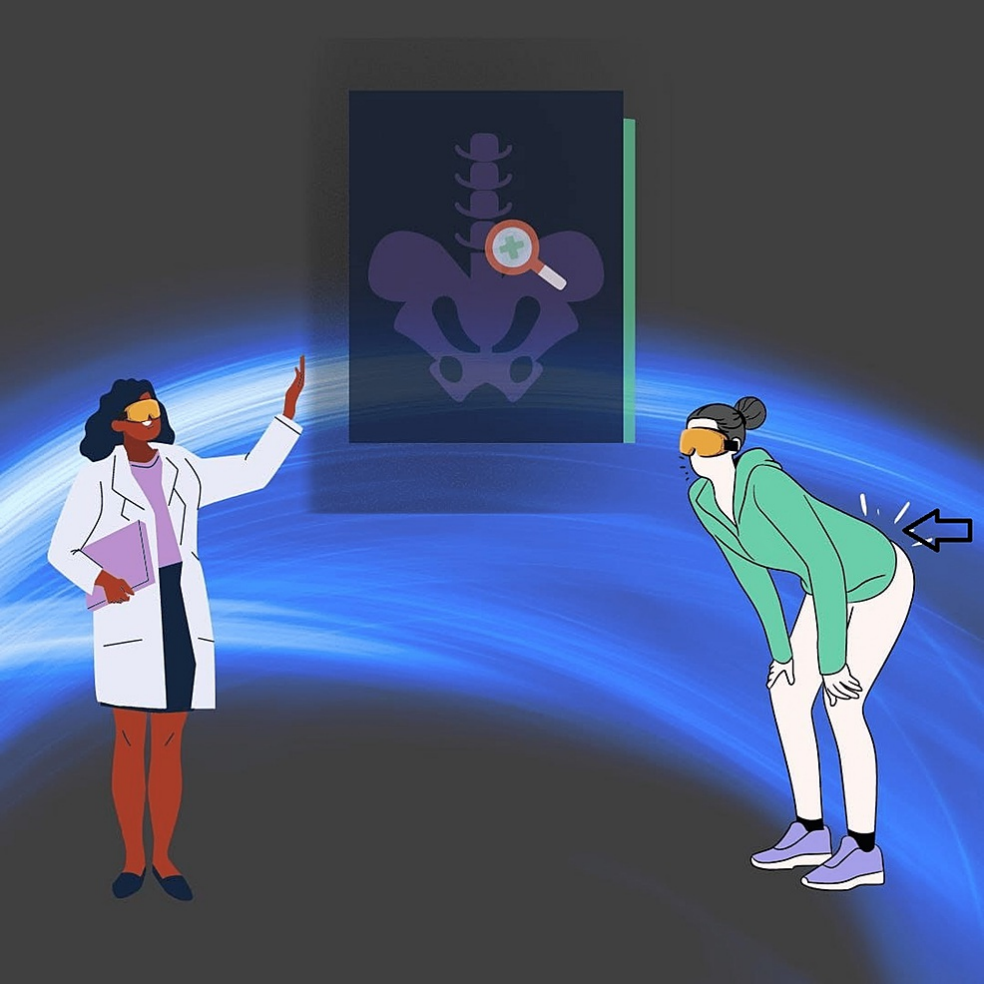
Representation of a doctor consultation via virtual reality Image illustrated by the author

It is obvious that the Metaverse is quite capable of linking real-time locations and objects utilized in the delivery of medical services and other virtual and physical things. The primary goal of the medical IoT is to complement traditional medical service delivery rather than replace it. It also aims to provide other routes that can digitally carry out physical tasks as effectively as possible when traditional methods are unavailable or inconvenient. First off, Metaverse will enhance both the effectiveness and the patient and physician experience of telemedicine (remote delivery of medical treatment). The Metaverse will make it possible for patients and physicians to interact virtually in real-time while in a virtual clinical setting using sensory teleportation items [17] (Figure 1). Some physical examination techniques such as distant body observation, touch, auscultation, and vital sign collection are permissible in this environment. Through the use of the Metaverse, sophisticated surgical procedures may be carried out digitally with high levels of vision and precision by human surgeons and surgeon robots [18]. 

Medical education

Medical education and training will be altered by the usage of AR and VR. Students can virtually enter into the human body, giving them a thorough perspective while allowing them to replicate real-life treatments. Augmented Reality is also being used to provide students with hands-on learning opportunities, such as mimicking patient and surgical contacts so that medical interns may envision and practice newer techniques. Even more realistic experiences based on real surgery might be built, allowing students to experience surgery as if they were the surgeon themselves [19]. Learning will be changed into an immersive experience in which success will be rewarded, and data analytics will be used to target precision learning.

**Figure 2:**
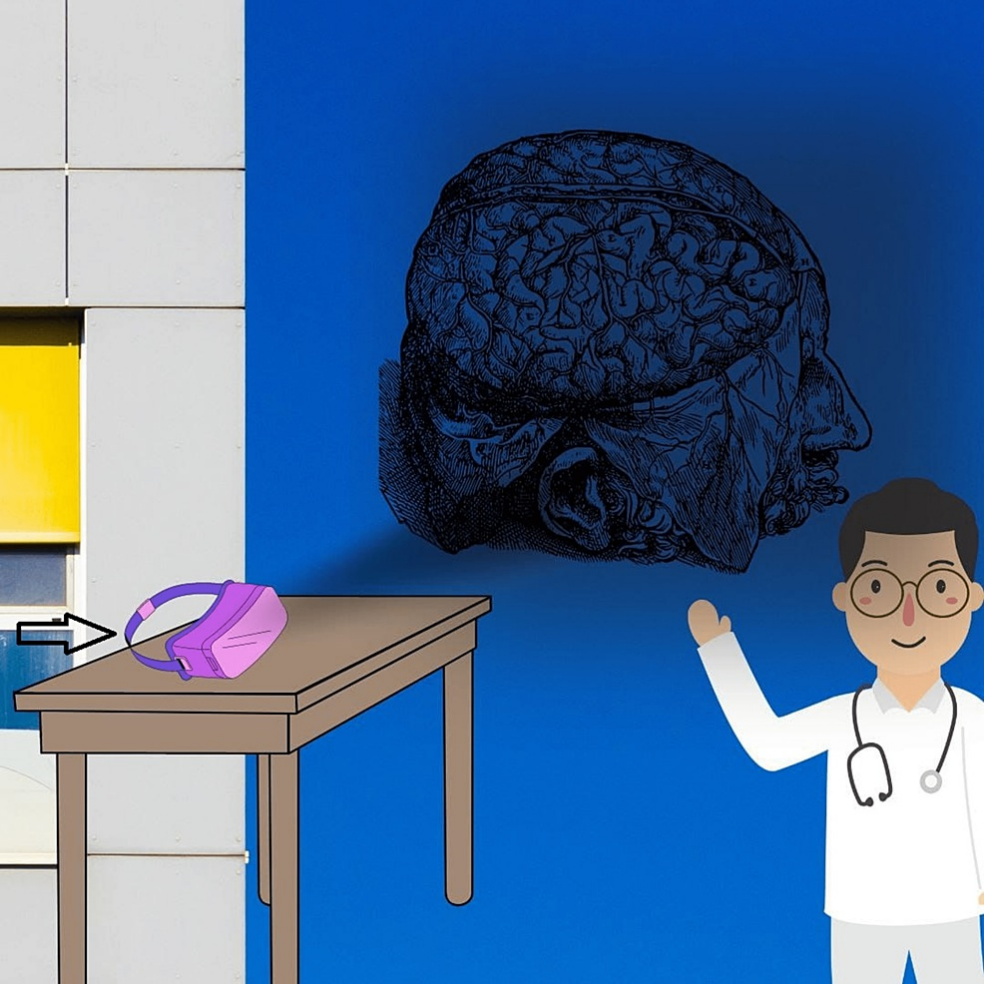
Representation of a live neurology class via virtual reality Image illustrated by the author

Since surgeries on cadavers are expensive for hospitals and have an impact on the students' tuition costs, the traditional medical school has limited resources for the practice of surgeries. But using VR in medical education allows students to train in a simulated setting for intensive surgical instruction at a substantially reduced cost [20] rather than just knowledge transmission (Figure 2). Advanced hand skills and interactions, for example, necessitate additional technology in Metaverse-based healthcare training, which is more successful. Surgical intervention, for example, necessitates a thorough mastery of human anatomy and dexterity in grasping equipment. This involves the employment of appropriate tracking devices or software [21]. 

As doctors always seek more effective ways to complete surgeries with a greater success rate, technology could also be used in the Metaverse for difficult surgical operations. Doctors may estimate a patient's recovery period, any potential difficulties, and the necessary therapies for these complications using the data set gathered by the patient's digital twin as a preventative strategy [20].

Instructors are responsible for providing high-quality data that can be used in virtual programs to imitate on-site nursing competencies. In a clinical field experience program, learners should feel that there was no difference in clinical therapy after accomplishing this in the Metaverse context. It is a significant addition to health care that is expected to improve patient care in the long run [21]. The study is a real-world example of gamified learning. For each class attended, each video watched, and each assignment completed, users are rewarded with tokens, and some will be rewarded with non-fungible token (NFT) collectibles. Artificial Intelligence educators can use AR to show students how to stand, speak, and appear more confident. Students will learn greatly in a truly game-like situation with popular trainers demonstrating specific abilities using these methodologies. This may be a celebrity surgeon, with the surgeon receiving compensation for his instruction and the student receiving compensation for his learning [22].

Challenges

However, putting this strategy into action will be a problem in and of itself. Current Metaverse rivals like Fortnight and Roblox, as well as Meta's Horizon Worlds and Microsoft's Mesh, are not compatible. One cannot transfer content gained, purchased, or made in Roblox to Horizon Worlds and vice versa, which goes against the Metaverse's original premise.

Medicine has traditionally been thought of as a human-to-human relationship. A patient's problem is initially discussed with a doctor. The doctor then determines the symptoms based on the patient's physiological data, such as emotional and physical responses, clinical data, and so on. Finally, the doctor determines which therapy option is best for the patient. Modern society has been revolutionized by the rise of big data and AI technology [8] which is not easy for everyone to digest. 

Since VR and AR information is primarily visual, the foundations are not established in the provision of medical services, which need the use of all five senses, including touch. Developers that conflate VR with the Metaverse add to the misunderstanding. There is no doubt that the Metaverse worldview will eventually replace many service domains. Change and convenience are also highly anticipated. Our medical professionals, on the other hand, must prioritize human dignity, respect for life, and affection for and treatment of the human body. To avoid human harm from the system, it will be required to thoroughly analyze all situations, such as policies, and prepare accordingly [23].

In the health Metaverse, there are still difficulties alongside opportunities. In the Metaverse, patient privacy and life safety raise numerous challenges and concerns. From a variety of angles, the health Metaverse will significantly alter medical practice. The Metaverse necessarily raises different concerns affecting the personal, public, and national security as a result of its enormous number of users and inventive connections. Inappropriate management of people's physical and mental health activities may jeopardize their health [8]. Not to mention, building the Metaverse in the early stage needs to consider protecting user privacy and physical and psychological safety. The Metaverse with massively connected devices and people, inevitably has significant loopholes in security, raising the question of what supervisory measures can ensure proper moral restraint. The technology stack of health Metaverse also shows the risks and difficulties of maintaining a system that cannot be compromised by hackers. Such risks threaten the personalized nature of the doctor-patient relationship [8].

The Metaverse also raises intriguing mental health concerns, such as the following [24]: VR dependency; transforming mental health therapy in general; and people suffering from psychosis, schizophrenia, sadness, or anxiety being harmed. Metaverse is currently mainly promoted by certain technology giants such as Facebook, Microsoft, and so on. At the time when Metaverse is ready, people inevitably accept all kinds of censorship and become victims of all sorts of commercial interests. Zhou et al. found that the design of the Metaverse business model is more biased towards platform owners, thereby weakening other competitors, which is often not conducive to the sustainable development of the platform [8].

Discussion

It is safe to say that our reality is now very much augmented. But the medical industry took this premise to a whole new level that surpassed even our most optimistic expectations [25]. Global Metaverse in the healthcare market is anticipated to exhibit a stunning compound annual growth rate (CAGR) of 33.7% to reach a market size of US$ 7453.6 Million by the end of 2028 during the forecast period of 2022 to 2028. The global market size for Metaverse in healthcare is worth US$ 5056.4 Million in 2021 [26]. The various advantages of this new technology include a three-dimensional (3D) visualization, better understanding, a safe and controlled environment, more accurate explanation, uses in medical education, better and automated operative procedures, surgical operations and lots more. With time we will be seeing more uses coming up. Disadvantages include but are not limited to very expensive equipment, risk of addiction, concerns over privacy and security, and effects on mental health [27-33] (Table 1).

**Table 1:** Advantages and disadvantages of virtual/augmented reality use in healthcare [27-33]

Advantages	Disadvantages
Variability and customization	Expensive
Numerous areas of implementation	Risk of dependency
Three-dimensional visualization	It cannot replace real-life practice
Positive psychological effect	Privacy and security
Controlled and safe environment	Still in an experimental stage

Instead of merely reading the lengthy details on the bottle, patients may use AR to observe how a medicine works in 3D right in front of their eyes. With the use of AR technology, lab personnel could see their tests. Workers might begin working in pharmaceutical factories without any hands-on training since the machine would instruct them on what to do and how to accomplish it [34].

As healthcare accelerates toward value-based care, the fast adoption of the Metaverse among healthcare providers remains a true possibility in the coming years. But before implementation, it is essential for healthcare providers to understand what patients require and how this innovative technology can serve an unmet need [35].

## Conclusions

It is difficult to test Metaverse programs because they are still in the experimental stage (determining and recording if a system regularly generates outcomes that satisfy the predetermined criteria). Hence, access to the Metaverse should be expanded rather than restricted. There are risks, but there are also a lot of opportunities. Using the technologically literate young population to take responsibility for their health care and be rewarded for learning, following wellness, and educating their peer groups in a safer social virtual place is a compelling proposal. Pioneers are transforming health education into engaging mini-courses that can be taught online to anybody, anywhere. The prospects for clinicians cooperating throughout the world and aided by AR give the opportunities to address professional health shortages. The opportunity to compensate the community, patients, and professionals for their endeavors to enhance their health opens up a completely new market and income opportunities. Stakeholders in the medical and health industry, such as doctors, patients, ordinary people, government decision-makers, and others, will benefit from the health Metaverse. The health Metaverse application can promote innovative medical education, surgery, medical treatment, and online health management.

This is a new universe that is evolving on a daily basis, and our understanding is expanding along with the pioneers and reformers who are creating these new Metaverses. It is possible to develop a sustainable and economical healthcare paradigm, and healthcare executives must be involved in the process. It is time to take a chance and see what possibilities exist.
